# Identification of compound heterozygous deletion of the WWOX gene in WOREE syndrome

**DOI:** 10.1186/s12920-023-01731-4

**Published:** 2023-11-16

**Authors:** Xing-sheng Dong, Xiao-jun Wen, Sheng Zhang, De-gang Wang, Yi Xiong, Zhi-ming Li

**Affiliations:** 1 Prenatal Diagnosis Center, Zhongshan Boai Hospital Affiliated to Southern Medical University, Zhongshan, Guangdong China; 2 Reproductive Medicine Center, Zhongshan Boai Hospital Affiliated to Southern Medical University, Zhongshan, Guangdong China; 3 Department of Pediatrics, Zhongshan Boai Hospital Affiliated to Southern Medical University, Zhongshan, Guangdong China

**Keywords:** WOREE syndrome, Epilepsy, *WWOX* gene, Discontinuous deletion, Breakpoints, Whole genome sequencing

## Abstract

**Background:**

Biallelic loss-of-function variants in WWOX cause WWOX-related epileptic encephalopathy (WOREE syndrome), which has been reported in 60 affected individuals to date. In this study, we report on an affected individual with WOREE syndrome who presented with early-onset refractory seizures and global neurodevelopmental delay and died at the age of two and a half years.

**Methods:**

We present clinical and molecular findings in the affected individual, including biallelic pathogenic variants in the *WWOX* gene. We employed different molecular approaches, such as whole exome sequencing, quantitative real-time polymerase chain reaction (qPCR), and whole-genome sequencing, to identify the genetic variants. The breakpoints were determined through gap PCR and Sanger sequencing.

**Result:**

Whole exome sequencing revealed homozygous exon 6 deletion in the *WWOX* gene in the proband. Quantitative real-time PCR confirmed that the parents were heterozygous carriers of exon 6 deletion. However, using whole-genome sequencing, we identified three larger deletions (maternal allele with exon 6–8 deletion and paternal allele with two deletions in proximity one in intron 5 and the other in exon 6) involving the *WWOX* gene in the proband, with deletion sizes of 13,261 bp, 53,904 bp, and 177,200 bp. The exact breakpoints were confirmed through gap PCR and Sanger sequencing. We found that the proband inherited the discontinuous deletion of intron 5 and exon 6 from the father, and the exons 6–8 deletion from the mother using gap PCR.

**Conclusion:**

Our findings extend the variant spectrum of WOREE syndrome and support the critical role of the *WWOX* gene in neural development.

**Supplementary Information:**

The online version contains supplementary material available at 10.1186/s12920-023-01731-4.

## Introduction

The WW domain-containing oxidoreductase (*WWOX*) gene maps to chromosome 16q23.1–23.2 and spans the second most common chromosomal fragile site (FRA16D) frequently altered in cancer [1, 2]. The *WWOX* gene encodes a 414-amino-acid protein consisting of two tandem WW domains in its N-terminus and an extended short-chain dehydrogenase/reductase (SDR) domain in its C-terminus [1, 3]. Initially, WWOX was discovered as a tumor suppressor [4, 5]. However, in recent years, animal models demonstrated that pathogenic variants of WWOX are associated with two major autosomal recessive neurological disorders, a relatively mild spinocerebellar ataxia 12 (SCAR12; OMIM:614322) and a more severe early infantile WWOX-related epileptic encephalopathy (WOREE syndrome). The WOREE syndrome is also known as developmental and epileptic encephalopathy 28 (EIEE28; OMIM:616211), depending on the type of mutation and its effect on WWOX expression [6, 7].

The clinical spectrum of WOREE syndrome includes early onset of refractory seizures, encephalopathy, spasticity with hyperreflexia and hypokinesia, developmental delay, structural brain abnormalities and premature death within the first years. Most of the affected individuals with WOREE syndrome make no eye contact and are not able to sit, speak or walk [6, 8]. Regarding SCAR12, only 6 affected individuals have been reported in two consanguineous families due to homozygous missense mutations. Affected individuals with SCAR12 display a milder phenotype, including early-onset seizures, delayed psychomotor development with intellectual disability and cerebellar ataxia. They can be treated with antiepileptic drugs and no premature death has been reported [6, 8]. Brain abnormalities were found especially in the WORRE syndrome, such as hypoplasia of the corpus callosum, progressive cerebral atrophy, delayed myelination and optic nerve atrophy [6, 9, 10]. Genotypes with a missense variation and a null allele resulted in an intermediate phenotype. Biallelic missense (hypomorphic) variants in the WWOX gene are associated with SCAR12, while two null alleles are associated with the WOREE syndrome [6].

The reported correlation between genotype and phenotype of these diseases is limited because of the scarcity of reported affected individuals in the literature. In this study, we reported that one affected individual had severe early-onset refractory seizures who had a compound heterozygous deletion composed of a discontinuous deletion of intron 5 and exon 6 in one allele and an exon 6 to 8 deletion on the other allele in the WWOX gene. We confirmed the exact breakpoints of these deletions using whole genome sequencing combined with gap polymerase chain reaction (PCR).

## Materials and methods

### Ethics approval and consent to participate

Blood samples were collected after informed consent had been obtained from the affected individual or their relatives. All procedures performed in studies involving human participants were in accordance with the 1964 Helsinki declaration and its later amendments or comparable ethical standards. The study was approved by the Ethics Committee of Zhongshan Boai Hospital Affiliated to Southern Medical University (protocol code KY-2021-008-03).

### Whole exome sequencing

The whole exome sequencing (WES) was performed as previously described [11]. Briefly, genomic DNA of the proband was extracted from peripheral blood. KAPA HyperExome Probes (Roche NimbleGen, USA) were used for capturing sequences. The enriched library was sequenced with the MGIseq-2000 platform using a paired-end 100 bp sequencing strategy. Raw reads were filtered according to previously published criteria [12]. Then, clean reads were mapped to the reference genome GRCh38 by using Burrows-Wheeler Aligner (BWA). Single-nucleotide variants (SNVs), insertions and deletions (indels) were called using the Genome Analyses Tool Kit (GATK) and annotated with SnpEff software. All candidate variants were filtered for data interpretation with minor allele frequency < 0.05 in dbSNP (https://www.ncbi.nlm.nih.gov/SNP), HapMap (https://www.genome.gov/international-hapmap-project), 1000 Genomes Project (www.internationalgenome.org) and a database of 100 healthy Chinese adults. Copy number variations (CNVs) were detected using the ExomeDepth R package. According to the American College of Medical Genetics (ACMG) guideline [13], all variants are classified as benign, likely benign, variants of unknown clinical significance (VUS), likely pathogenic and pathogenic.

### Quantitative real-time PCR

To confirm the deletion involving exon 6 of the *WWOX* gene, we developed a gene dosage assay based on quantitative real-time PCR (qRT-PCR). DNA samples from all family members and a healthy control were diluted in 50 ng/μl. The qRT-PCR was conducted in a total of 20 μl containing 10 μl 2× GoTaq® qPCR Master Mix (Promega, USA), 0.25 μl of each primer pairs (10 μM), 1 μl of DNA sample (50 ng/μl) and ddH_2_O. The PCR reaction was performed using the SLAN-96S real-time PCR System (Hongshi Med. Tec., China). The cycling conditions were as follows: 95 °C for 2 min, then 40 cycles of 95 °C for 15 s and 60 °C for 20 s. The WWOX-exon6 primer pairs were generated as forward primer (5′-TACCATGAACTACACTTGCTGT-3′) and reverse primer (5′-GATCTATAACCCTCCACTGGAAC-3′). The *GAPDH* gene was used as an internal reference and primer pairs were generated as forward primer (5′- GTCAGTGGTGGACCTGACCT-3′) and reverse primer (5′- TCGCTGTTGAAGTCAGAGGA-3′). The fold-change was calculated using the 2^-ΔΔCt^ method.

### Whole genome sequencing

In order to confirm the deletions and accurately identify their breakpoints, whole genomic sequencing (30× WGS) was performed on the proband. The qualified genomic DNA was randomly fragmented by Covaris sonicator and the proper fragment (350 bp) was obtained after fragment selection. The WGS libraries were prepared by MGIEasy FS DNA Prep kit (BGI, China) according to the manufacturer’s instructions. Then, they were sequenced using DNBSEQ-T7 platforms generating 2 × 100 bp paired-end reads. The WGS data were processed using an in-house analysis pipeline. Briefly, the raw data quality check was conducted using FastQC (version 0.11.9). Reads were.

mapped to the reference genome (GRCh38) using BWA-MEM (version 0.7.17-r1188) and GATK (version 4.2.1.0). Duplicates were removed using Picard MarkDuplicates (version 2.21.2). The CNVs were called using the CNVnator (version 0.2.7) read-depth algorithm. The structural variations (SV) were detected using the BreakDancer-1.3.6.

### Gap PCR analysis

Deletion breakpoints in the *WWOX* gene were confirmed by gap PCR and Sanger sequencing. PCR primers flanking the breakpoints were generated as follows: F1 (5′-TTTCTGGAGCAGTCTATTT-3′) and R1 (5′-TGAACAGCCAGCCAATAC-3′) for the deletion in exons 6–8; F2 (5′-AGGGTATTAACATCTTGCA-3′) and R2 (5′-ATCTGCTCCGCTTAGTCA-3′) for the deletion in the intron 5; F3 (5′-TAAGGGCTCAGTAGCGTAG-3′) and R3 (5′- CAAATCTACCGACCTTAT-3′) for the deletion in exons 6. The PCR products were analyzed by electrophoresis in agarose gel (1.2%) and specific fragments were sequenced. Sequencing results were queried online using the UCSC Genome Browser tool (Human GRCh38/hg38, https://genome.ucsc.edu) to identify breakpoints. After identifying breakpoints, sequences flanking the breakpoints were screened for repetitive elements using the RepeatMasker program (http://www.repeatmasker.org/) to identify repetitive sequences at breakpoint junctions. The R-loop forming sequences (RLFSs) were screened by R-loopDB (http://rloop.bii.a-star.edu.sg).

## Results

### Clinical features

**Fig. 1:**
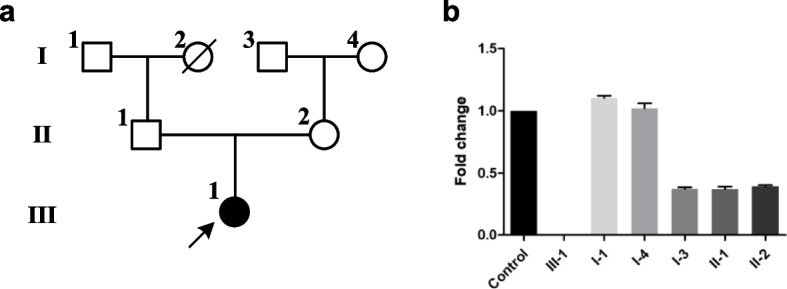
**a** Pedigree of the family. The proband is labeled with arrow. **b** Quantitative PCR validation of the exon 6 deletion of the *WWOX* gene. The Y-axis represents the log R ratio, the X-axis indicates the exon 6

The affected individual was born at term by normal spontaneous vaginal delivery, the weight was 3.39 kg (birth weight percentile 50%). No history of hypoxia during the perinatal period was reported. At 15 days after birth, he presented infantile seizures characterized by clenched teeth, cyanosis around the lips and tetanic twitch of the limbs.. He seizure lasted and he continued to have seizures with a frequency of 10–15 times per day. The administration of antiepileptic drugs (sodium valproate and topiramate tablet) was not effective. He did several hospital admissions because of recurrent seizure attacks. The affected individual growth retardation and developmental delay and was unable to follow objects, roll over, sit alone or speak. Electroencephalograms (EEGs) shows slow weakened of background activity observed in both hemispheres and and polyspikes low-wave discharge in bilateral temporal lobes. Magnetic resonance imaging (MRI) showed white matter hyperintensity and delayed myelination in the brain. He died at the age of two and a half years because of persistent seizures. At the time of death, the affected individual was 69 cm (−6SD) in height and 8 kg (−4SD) in weight. The familiar medical history was unremarkable except for the maternal grandfather (I-3) who suffered from lung cancer and the paternal grandmother (I-2) who died of a cerebrovascular accident more than 10 years ago (Fig. 1a).

### Molecular findings

The WES analysis revealed a homozygous deletion involving exon 6 of the *WWOX* gene in the proband. The qRT-PCR indicated that the maternal grandfather (I3), father (II1) and mother (II2) were characterized by a heterozygous deletion involving exon 6 of the *WWOX* gene (Fig. 1b). No amplification was seen in the proband as if he harbors a biallelic deletion of exon 6.

**Fig. 2:**
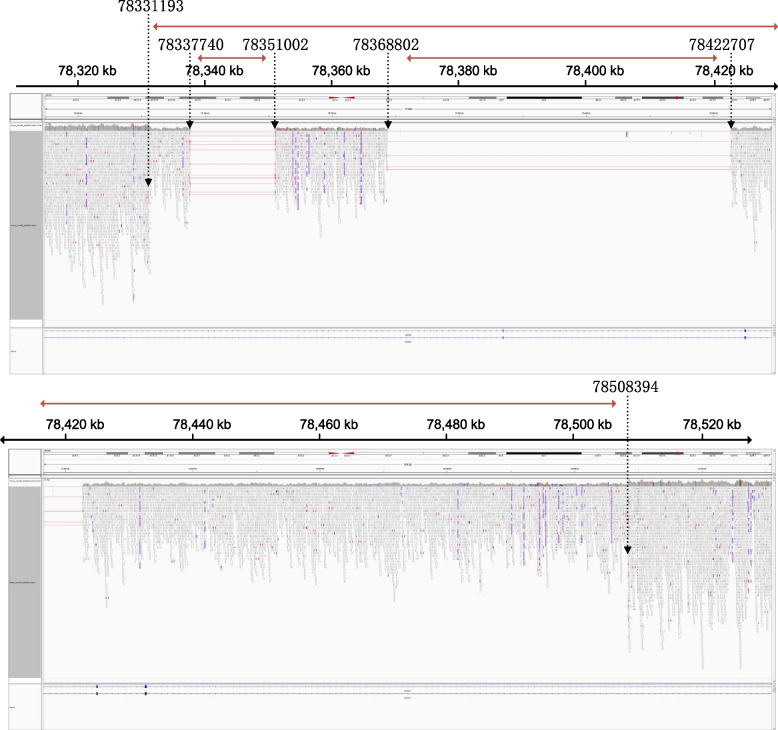
Whole genome sequencing confirmed the deletions of the *WWOX* gene. The horizontal red lines in the red alignments represent deletions in alignments that otherwise match the reference sequence. Breakpoints can be inferred from the short reads by examination of the outer read extents and the depth in coverage. The black dotted arrow represents the breaking point, the red double arrow line represents the range of deletions

Interestingly, the WGS analysis revealed also the presence of three larger deletions in the *WWOX* gene in the proband, not two deletions (Fig. 2). A large deletion about 177,200 bp in length involved exons 6–8 at chr16: 78331193–78,508,394 (hg38). Another deletion about 13,261 bp in length involved intron 5 at chr16: 78337740–78,351,002 (hg38). The last deletion about 53,904 bp in length involved exon 6 at chr16: 78368802–78,422,707 (hg38).

**Fig. 3:**
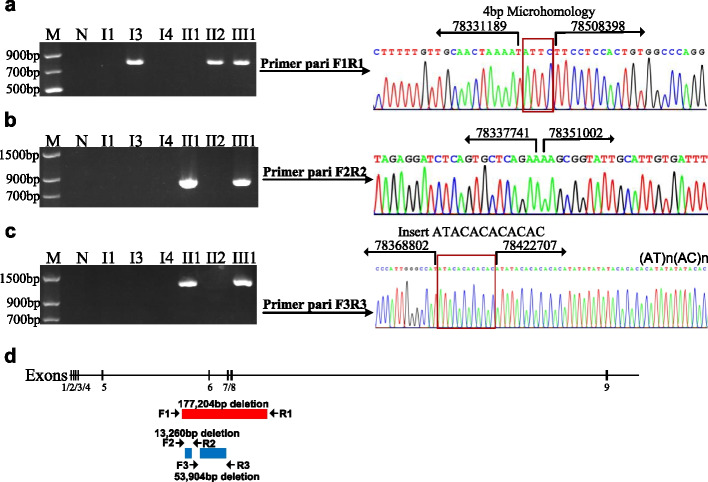
Identification of the breakpoints of the three deletions. **a** A 800-bp PCR product was amplified by gap-PCR using primers F1 and R1 in I3, II2 and III1. No PCR product was observed from I1, I4, II1 or the control. The sequencing results showed that the deletion junction was characterized by a 4-bp microhomology. The 5′ breakpoint was located within coordinates 78,331,189 and 78,331,193, the 3′ breakpoint was located within coordinates 78,508,394 and 78,508,398. M: marker, N: normal individual. **b** A 900-bp PCR product was amplified by gap-PCR using primers F2 and R2 in II1 and III1. No PCR product was observed from I1, I3, I4, II2 or the control. The sequencing results showed that the 5′ breakpoint was located at 78337741, the 3′ breakpoint was located at 78351002. **c** A 1300-bp PCR product was amplified by gap-PCR using primers F3 and R3 in II1 and III1. No PCR product was observed from I1, I3, I4, II2 or the control. The sequencing results showed that the 5′ breakpoint was located at 78368802, the 3′ breakpoint was located at 78422707. The deletion junction inserted ATACACACACAC. The 3′ flanking regions were characterized by (AT)n(AC)n repetitive sequences. **d** Schematic representation of the three deletions. The deletion inherited from proband’s mother is represented as a red bar. The other two deletion are represented as blue bars

The gap PCR and Sanger sequencing were performed to identify three deletions. In I3, II2, and III1, we found a 177,204 bp deletion (exons 6–8 deletion) with breakpoints between 78,331,189–78,331,193 and 78,508,394–78,508,398 (Fig. 3a, d). In II1 and III1, there was a 13,260 bp deletion (intron 5 deletion) with breakpoints at 78337741 and 78,351,002 (Fig. 3b, d). In II1 and III1, a 53,904 bp deletion (exons 6 deletion) was detected with breakpoints at 78368802 and 78,422,707, along with an ATACACACACAC insertion at the deletion junction. The 3′ flanking regions were (AT)n(AC)n repetitive sequences (Fig. 3c, d). The exons 6–8 deletion (177,204 bp) was inherited from the mother (II2), while the discontinuous deletion of intron 5(53,904 bp) and exon 6(13,260 bp) were inherited from the father (II1).

## Discussion

The clinical phenotypes associated with germline, bi-allelic and pathogenic variants of the *WWOX* gene are highly heterogeneous. Symptoms range from a mild phenotypic SCAR12 to a severe early infantile WWOX-related epileptic encephalopathy (WOREE syndrome), depending on the type of mutation and its effect on WWOX expression. Therefore, accurate diagnosis and treatment are hampered by the heterogeneous clinical presentation of individuals. WOREE syndrome is characterized by early onset, epilepsy, growth retardation, delayed psychomotor development, and progressive microcephaly. Early-onset epilepsy is a core feature of the neurological phenotype of WOREE syndrome, which is associated with a high premature death rate [14]. In this study, we describe a child from an unrelated Chinese family who presented with growth retardation, early seizure disorder at birth, and followed by global developmental delay. Our proband had the earliest onset of epilepsy, presenting on the 15th day of life. Unfortunately, his multidaily tonic seizures never responded to any pharmacological treatment, and he died at the age of two and a half years due to persistent seizures. Our proband’s phenotype was very similar to the affected individual described by Abdel-Salam et al., who had a homozygous loss-of-function germline variation in the *WWOX* gene [15].

In this study, we identified a proband with a total of three deletions with different breakpoints in WWOX, one deletion involving exons 6 and 8 on the maternal allele and the other two as discontinuous deletion on the paternal allele. The intron 5 and exon 6 deletions were found in a single allele, and were discontinuous. Even though deletions on both alleles were previously reported, the combination of them in a compound heterozygous state resulting in this complex germline structural variation has not been previously encountered. The germline intron 5 deletion is regarded as a polymorphism but also has been associated with several cancers in the Han Chinese (Hussaine et al., 2019) [2]. The exon 6 deletion (NM_016373.3: c.517_605del, His173AlafsTer67) is a common deletion predicted to cause a frameshift mutation resulting in a truncated protein with loss of function. In our proband, the exon 6–8 deletion was found in the other allele of the *WWOX* gene. The exon 6–8 deletion (NM_016373.3:c.517_1056del, His173_Met352del) theoretically produces a protein with normal WW domains, but due to the disruption of the SDR domain, it may retain poor residual function [10]. Mignot et al. reported a case of an affected individual with WOREE syndrome who had compound heterozygous deletions in *WWOX gene* that encompassed exon 1–5 and exon 6–8 t^10^. Similarly, in our case, compound heterozygous deletions were found to be the cause of WOREE syndrome, which is consistent with the proposed mechanism of the syndrome. The proband’s clinical phenotype closely mirrors what has been previously reported in the literature regarding exon 6 deletion or exons 6–8 deletion, resembling WOREE syndrome. Previous reports have provided detailed descriptions of the clinical phenotypes (see Supplementary Table 1). In 2009, Suzuki et al. found that the pathogenic variant of *WWOX* was associated with epileptogenesis in mice [16]. However, the pathophysiological mechanism underlying the development of WOREE syndrome due to total or partial loss of function of WWOX has yet to be fully understood. In-depth analysis of WWOX function in the brain is ongoing. Studies in the *WWOX* knockout mouse model have demonstrated spontaneous bursts of activity in the neocortex, suggesting that WWOX plays a fundamental role in balancing neocortical excitability [17].

WWOX is a hot spot for both germline and somatic copy number variants. The WWOX gene encompasses FRA16D, spanning from introns 5 to 8, indicating its susceptibility to breakages and rearrangements. Multiple models have tried to explain the underlying mechanisms behind the FRA16D instability, including longer AT repeats forming a cruciform and stall replication. Replication origins located within common fragile sites (CFS) sequences are less efficient and probably responsible for replication perturbation along fragile sites [2, 18–20] An exon 6 deletion and an intron 5 deletion were both found on a single allele in this study, suggesting that the chromosome underwent two separate breakage and rearrangement events. . In order to explain how WWOX deletions involving FRA16D were generated, we used 30× WGS combined with PCR to identify the breakpoints. We accurately identified the breakpoint junctions of three deletions. No R-loop forming sequences were found using R-loopDB to screen the sequences flanking the breakpoint junctions of the three deletions. The deletion involving exons 6–8 and another deletion involving intron 5 were presumably generated by the non-homologous end joining (NHEJ) repair pathway, because this model requires one to four base pairs or no homology between proximal and distal breakpoints. The 3′ flanking regions were (AT)n(AC)n repetitive sequences involving exon 6. The deletion might be caused by the abundance of AT-rich DNA forming complex secondary structures that slow down or block the progress of the replication machinery and ultimately leading to chromosome breakage [19].

WWOX has been extensively studied for its role in cancer because it localizes to a common fragile site, FRA16D, a genomic region susceptible to chromosomal rearrangements. Different in vitro and in vivo functional studies have indicated WWOX’s role as a tumor suppressor. The reduction or absence of WWOX expression has been associated with several cancers, including breast cancer, thyroid cancer, oral cancer and lung cancer [21]. WWOX expression is altered by deletion and/or aberrant synthesis in a significant number of non-small cell lung cancer (NSCLC) tumors (51.8%) [22]. Moreover, a high incidence of exons 6–8 deletions has been reported in Chinese affected individuals with NSCLC (63.6%), suggesting that exons 6–8 deletions might play a role in the tumorigenesis of NSCLC [23]. In this study, we hypothesized that the heterozygous *WWOX* exons 6–8 deletion observed in maternal grandfather (I-3, lung cancer affected individual) caused an abnormal WWOX expression and the consequent clinical phenotype.

In conclusion, we reported a compound heterozygous deletion composed of a discontinuous deletion of intron 5 and exon 6 in one allele and an exon 6 to 8 deletion on the other allele in the WWOX gene, which causing a more severe early infantile WOREE syndrome. Furthermore, we confirmed the exact breakpoints of these deletions using WGS combined with gap PCR. Our findings extend the mutation spectrum of the WOREE syndrome and support an important role for the *WWOX* gene in neural development. Genetic testing is essential for both establishing a definitive diagnosis and offering genetic counseling for WOREE syndrome. While WES may encounter some pitfalls in detecting copy number variations in the WWOX gene, WGS accurately addresses intricate variant configurations and investigates potential mechanisms underlying their formation.

### Supplementary Information

**Additional file 1.**

**Additional file 2. **

## Data Availability

The data that support the findings of this study have been deposited into CNGB Sequence Archive (CNSA) [24] of China National GeneBank DataBase (CNGBdb) [25] with accession number CNP0003486. http://db.cngb.org/cnsa/project/CNP0003486_87bbbaec/reviewlink/.
