# Supplementary material for: Identification of compound heterozygous deletion of the WWOX gene in WOREE syndrome
Source: BMC Med Genomics. 2023 Nov 16;16:291. doi: 10.1186/s12920-023-01731-4 (PMC10652538; doi:10.1186/s12920-023-01731-4)
Supplement: Supplementary file 1 — Additional file 1. [file 12920_2023_1731_MOESM1_ESM.docx]

|  | **This study** | **Mignot C,**  **et al. 2015**  **-Patient1** | **Mignot C,**  **et al. 2015**  **-Patient2** | **Davids M,**  **et al. 2019** | **Riva A, et al. 2022** |
| --- | --- | --- | --- | --- | --- |
| **Gender** | Male | Female | Female | Female | Male |
| **hypotonia** | Yes | Yes | Yes | Yes | Yes |
| **Psychomotor Delay** | Yes | Yes | Yes | Yes | Yes |
| **Epilepsy (onset)** | 15 days | 2 months | 2 moths | 2 weeks | 1 day |
| **Seizures types** | Generalised clonic seizures | Focal tonic seizures | Generalised clonic and tonic  unilateral brachiofacial | tonic-clonic and myoclonic seizures | Asymmetric tonic seizures |
| **EEG**  **pattern** | slow weakened of background activity observed in both hemispheres and and polyspikes low-wave discharge in bilateral temporal lobes | Disorganised slow background activity,  occipital slow waves and  paroxysm | Disorganised slow background activity, bioccipital  sharp and slow waves,  bioccipitotemporal seizures | Awake EEG showed diffuse delta frequencies at approximately four hertz. In drowsy states, background activity was a mixture of delta frequencies. No significant sleep architecture was seen in sleep phases. The spike-and-wave discharges and bursts of spike-and-waves are decreased during sleep. | Slow monomorphic activity, multifocal independent slow wave |
|  | **This study** | **Mignot C.**  **et al. 2015**  **-Patient1** | **Mignot C.**  **et al. 2015**  **-Patient2** | **Mariska Davids.**  **et al. 2019** | **Antonella Riva, et al. 2022** |
| **Response**  **to Anti-seizure medications** | Drug Resistant | Partial | Partial | Drug Resistant | Drug Resistant |
| **Brain MRI** | white matter hyperintensity and delayed myelination in the brain | thin corpus callosum | Mild myelination delay | moderate cortical atrophy, thin corpus callosum, minor posterior thinning of the cerebellar vermis, enlargement of ventricles, and prominent cisterns | Brain atrophy, thin CC, inferior cerebellar vermis hypoplasia, periventricular leukomalacia-like pattern |
| **Premature**  **death** | two and a half years | No | 16 moths | No | No |
| **Mutations** | the discontinuous deletion of intron 5 , exon 6 and the exons 6-8 deletion of WWOX | exons 1–5 deletion and exons 6–8 deletion of WWOX | exon 6 deletion and nonsense exon 8 of WWOX | homozygous exon 6 deletion of WWOX | Stop gained variant and exon 6 deletion of WWOX |
