# Supplementary material for: Identification of compound heterozygous deletion of the WWOX gene in WOREE syndrome
Source: BMC Med Genomics. 2023 Nov 16;16:291. doi: 10.1186/s12920-023-01731-4 (PMC10652538; doi:10.1186/s12920-023-01731-4)
Supplement: Supplementary file 2 — Additional file 2. [file 12920_2023_1731_MOESM2_ESM.docx]

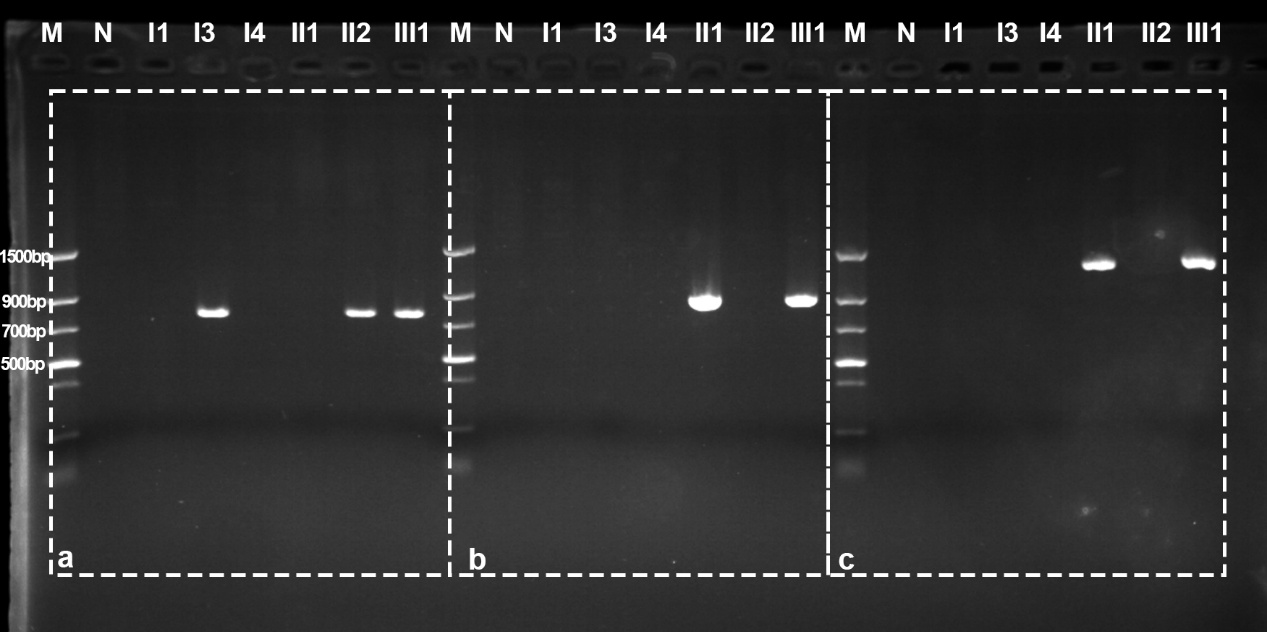


Supplementary Figure 1：This is the original figure of Figure 3 in the manuscript. **a**. A 800-bp PCR product was amplified by gap-PCR using primers F1 and R1 in I3, II2 and III1. No PCR product was observed from I1, I4, II1 or the control. M: marker, N: normal individual. **b**. A 900-bp PCR product was amplified by gap-PCR using primers F2 and R2 in II1 and III1. No PCR product was observed from I1, I3, I4, II2 or the control. **c**. A 1300-bp PCR product was amplified by gap-PCR using primers F3 and R3 in II1 and III1. No PCR product was observed from I1, I3, I4, II2 or the control.
